# New taxonomic and conservation status of *Ossiculum* (Vandeae, Orchidaceae), a highly threatened and narrow-endemic angraecoid orchid from Central Africa

**DOI:** 10.3897/phytokeys.98.23511

**Published:** 2018-05-02

**Authors:** Murielle Simo-Droissart, Tariq Stévart, Bonaventure Sonké, Sandrine Mayogo, Narcisse Kamdem, Vincent Droissart

**Affiliations:** 1 Plant Systematics and Ecology Laboratory, Higher Teachers’ Training College, University of Yaoundé I, P.O. Box 047, Yaoundé, Cameroon; 2 Missouri Botanical Garden, Africa and Madagascar Department, P.O. Box 299, St. Louis, Missouri 63166-0299, U. S. A; 3 Herbarium et Bibliothèque de Botanique africaine, C.P. 265, Université Libre de Bruxelles, Campus de la Plaine, Boulevard du Triomphe 1050, Brussels, Belgium; 4 Botanic Garden Meise, Domein van Bouchout, Nieuwelaan 38, B-1860 Meise, Belgium; 5 AMAP, IRD, CIRAD, CNRS, INRA, Univ Montpellier, Montpellier, France

**Keywords:** Angraecoid orchids, *Calyptrochilum*, *ex situ* conservation, IUCN Red List Categories and Criteria, Mungo River Forest Reserve, Odzala National Park, *Ossiculum aurantiacum*

## Abstract

In the context of producing a revised phylogenetic Linnean taxonomy of angraecoid orchids, the monotypic and narrow-endemic genus *Ossiculum* is synonymised with *Calyptrochilum*. Accordingly, a new combination in *Calyptrochilum* is proposed for *Ossiculum aurantiacum*. The morphological and DNA-based evidence for this transfer is discussed. Moreover, *Calyptrochilum aurantiacum* is here firstly reported outside Cameroon, with a record from the Republic of the Congo. The Red List conservation status of this species is reassessed and it is to be downgraded from “Critically Endangered” (CR) to “Endangered” (EN), following the recent discovery of additional subpopulations in Cameroon.

## Abstract (fr)

En vue de produire une classification taxonomique linnéenne des orchidées angraecoïdes, le genre monotypique *Ossiculum*, longtemps considéré comme endémique du sud-ouest Cameroun, est placé en synonymie de *Calyptrochilum*. En conséquence, une nouvelle combinaison dans *Calyptrochilum* est proposée pour *Ossiculum aurantiacum*. Les données morphologiques et moléculaires qui justifient cette combinaison sont discutées. L’espèce est signalée pour la première fois hors du Cameroun, en République du Congo. Suite à la découverte récente de sous-populations supplémentaires au Cameroun, le statut de conservation de cette espèce est réévalué selon la liste rouge de l'UICN. Bien que toujours menacée, l’espèce *Calyptrochilum aurantiacum* est transférée de la catégorie “En danger critique” (CR) vers la catégorie “En danger” (EN).

## Introduction

While describing the monotypic genus *Ossiculum* P.J.Cribb & Laan, van der Laan and Cribb (1986) were the first to point out its morphological resemblance to *Calyptrochilum* Kraenzl, an angraecoid genus comprising two species widespread in tropical Africa. In fact, both genera possess a sigmoid clavate spur and their column bears a somewhat elongate bifid rostellum. Moreover, *Ossiculum aurantiacum* P.J.Cribb & Laan and *Calyptrochilum christyanum* (Rchb.f.) Summerh. have a yellow lip and a spur of similar shape. Later, Gasson and Cribb (1986), when describing the leaf anatomy of *O. aurantiacum*, found that *Ossiculum* and *Calyptrochilum* share an array of unique features which sets them apart from other angraecoid orchids: the same epidermal cell arrangements, spiral thickenings on some mesophyll cells, the presence of water storage cells, along with the absence of hypodermal and mesophyll sclereids and that of a palisade layer. By pooling the anatomical evidence with morphological and cytological data, these authors concluded that *Ossiculum* is most closely related to *Calyptrochilum*. The study of Arends and van der Laan (1986) on the cytology and morphology of angraecoid orchids recognised four groups based on rostellum length and basic chromosome number. One of these groups (the third one) comprised only *Calyptrochilum* and *Ossiculum* (rostellum elongated, x = 17 or 18). Twenty years later, the study of Carlsward et al. (2006) on comparative vegetative anatomy and systematics of 142 angraecoid orchid species also showed that *Ossiculum aurantiacum* and the two *Calyptrochilum* species have a very similar leaf anatomy. However, with its brightly yellow-orange flowers, its rostellum lobes resembling pincers and with a bipartite basal callus on the lip, *Ossiculum* is readily distinguished from the two species of *Calyptrochilum*, which bear predominantly white and green flowers, straight rostellum lobes and a three-lobed lip lacking a basal callus. In addition, the latter two species have a horizontal to pendent habit (vs an erect habit in *Ossiculum*) with the leaves more or less flat and arranged in a single plane (vs leaves V-shaped in cross-section).

*Ossiculum aurantiacum* was first discovered in the Mungo River Forest Reserve, Cameroon in 1980. At that time, the only specimens known were the type and a young seedling, both collected by the Dutch botanist H. J. Beentje. The young seedling flowered in cultivation in November 1983 at the University of Wageningen (The Netherlands) from which a voucher was collected (*van der Laan 718*). During the next 20 years, *O. aurantiacum* was not seen again despite the several thousand botanical collections that were made within 50 miles of the type locality (Pollard 2011). Based on that unique collection, the species was assessed as “Critically Endangered” (CR) on the IUCN Red List by Pollard and Darbyshire (2004).

In 2004, a new locality for this species was discovered in the Banyang-Mbo Wildlife Sanctuary, Cameroon. This wildlife sanctuary is widely held to be one of the most biologically important forest complexes in West Central Africa, harbouring 325 documented bird species, 71 amphibians, 63 reptiles and 33 large mammals (Riley and Riley 2005) and several narrow endemic plants (e.g. Cheek 2002; Sonké et al. 2002). Several large-scale anthropogenic disturbances (e.g. oil palm plantations in the west, timber exploitation in the north) have taken place around the sanctuary, menacing its biodiversity, notably the narrow range species which occur within the protected area and its surroundings (Asaha and Deakin 2016). The discovery of this new specimen in an additional location in Cameroon cast away the fears that *O. aurantiacum* might already be extinct and has fostered new specific surveys and a conservation programme dedicated to this species.

Thanks to intensive fieldwork in Cameroon in 2011 and 2017, we discovered new localities of *O. aurantiacum.* Newly collected specimens of the species were included into a phylogenetic study of African angraecoid orchids (Simo-Droissart et al. 2018a). In the meantime, examination of dried material deposited in BRLU (herbarium acronym according to Thiers 2018) led one of us (V. Droissart) to discover that the species has been collected in the Republic of the Congo in 1996, but had been misidentified as *Cyrtorchis* sp. This finding, along with new collections made at the type location in Cameroon in 2017, prompted us to reassess the IUCN conservation status of the species.

Based on these new data, this paper aims to (i) reappraise the taxonomy of *Ossiculum aurantiacum* and its generic status, (ii) update its distribution in light of the new collections in Cameroon and the first record of this species for the Republic of the Congo and (iii) define its ecology and threats and reassess its IUCN conservation status.

## Material and methods

On-site ecology and conservation status of *Ossiculum aurantiacum* were investigated during three main field campaigns in the Southwest Region of Cameroon (2004, 2011 and 2017). To look for additional collections, herbarium specimens of BR, BRLU, K, WAG and YA (herbaria acronyms according to Thiers 2018) were examined. The geographical distribution of the species was determined from data given on the herbarium sheets. New country records were identified by comparing the species distribution with the information provided by Govaerts et al. (2017). The species distribution map was prepared using ArcMap 10.4.1 (ESRI 2016).

Phylogenetic relationships between African angraecoid orchids, including *Ossiculum* and *Calyptrochilum*, were inferred on the basis of DNA sequence data from three regions (ITS, *matK*, and the *trnL-trnF* intergenic spacer) and 555 accessions representing 316 species from 43 genera (see Simo-Droissart et al. 2018a). We performed the parsimony and the Bayesian analyses on the combined matrix using, respectively, the computer programme PAUP* v.4.0 a 146 (Swofford 2002) and MrBayes 3.2.5 (Ronquist and Huelsenbeck 2003; Ronquist et al. 2012).

Using the IUCN Red List Categories and Criteria (IUCN 2012), we made a preliminary risk of extinction assessment for the species. We imported georeferenced specimen data into the *ConR* package (Dauby et al. 2017) to calculate the area of occupancy (AOO) and extent of occurrence (EOO). The cell size for AOO was set 2 × 2 km as recommended by IUCN (2017). We calculated the number of ‘locations’ (as defined by IUCN 2017) with regard to the kind of threats, such that a single ‘location’ may encompass more than one adjacent population.

## Results

### Taxonomy

Simo-Droissart et al. (2018a) included *Ossiculum aurantiacum* in their molecular study of African angraecoid orchids and showed that, in both parsimony and Bayesian analyses, *O. aurantiacum* was sister to the two species of *Calyptrochilum*. This relationship is supported with strong bootstrap (BS 100) and posterior probability (PP 1), while the sister relationship of the two *Calyptrochilum* species is supported moderately and only by the Bayesian analysis (PP 0.77). Given the morphological and cytological similarities between *Ossiculum* and *Calyptrochilum* (see above) and the molecular data, we consider the monotypic genus *Ossiculum* as a junior synonym of *Calyptrochilum* and propose the following amended generic diagnosis.

#### Calyptrochilum

Taxon classificationPlantaeAsparagalesOrchidaceae

Kraenzl., 1895: 30

Ossiculum van der Laan & Cribb, 1986: 823. Type species: Ossiculum aurantiacum van der Laan & Cribb, 1986: 824, **syn. nov.**

##### Type species.

*Calyptrochilum preussii* Kraenzl. (1895: 30) (= *Calyptrochilum emarginatum* (Afzel. ex Sw.) Schltr. (1918: 84)).

##### Basionym.

*Limodorum emarginatum* Afzel. ex Sw.(1805: 86).

##### Description.

Epiphytic or lithophytic herbs. Stem cylindrical, erect, spreading or pendent, covered by sheathing leaf bases. Leaves coriaceous, distichous, imbricate, unequally bilobed at tip, articulate to sheathing at the base. Inflorescence axillary, few- to many-flowered, shorter than leaves; bract distichous, cucullate. Flowers white, sometimes with a green or yellow mark on labellum or bright orange with a yellow labellum. Sepals ovate-elliptic, acuminate. Petals oblanceolate, acute. Labellum entire or trilobed, spurred at base; spur geniculate in middle, clavate at tip. Column with two pollinia, pollinia attached to an ovate or horseshoe-shaped viscidium.

##### Distribution and ecology.

*Calyptrochilum* is a genus of three species distributed throughout tropical Africa, from sea level to 1200 m and found as epiphyte in humid evergreen forests, humid woodland, savannahs and as lithophyte.

#### Calyptrochilum aurantiacum

Taxon classificationPlantaeAsparagalesOrchidaceae

(P.J.Cribb & Laan) Stévart, M.Simo & Droissart comb. nov.

urn:lsid:ipni.org:names:60476300-2

##### Basionym.

*Ossiculum aurantiacum* van der Laan & Cribb, 1986: 824.

##### Type.

Cameroon. Mungo River Forest Reserve, 13 km on road from Kumba to Loum, 04.682°N, 09.533°E, 16 Dec. 1980, *Beentje 1460A* (holotype: WAG! isotypes: K!, WAG!, YA!).

##### Additional specimens examined.

CAMEROON. Mungo River Forest Reserve, 13 km on road from Kumba to Loum, 04.682°N, 09.533°E, 16 Dec. 1980, *van der Laan 718* (WAG). Southwest Region, Nguti village, path between the WCS station and the camp “552”, outside of the Banyang-Mbo Wildlife Sanctuary, 05.337°N, 09.473°E, 15 November 2004, *Yaoundé shadehouse series 196* (BRLU, YA). *Ibid.*, 10 May 2005, *Yaoundé shadehouse series 265* (BRLU). *Ibid*., 20 September 2006, *Yaoundé shadehouse series 428* (BRLU). *Ibid*., inside of the Banyang-Mbo Wildlife Sanctuary, 05.344°N, 09.517°E, 9 May 2011, *Yaoundé shadehouse series 2773* (BRLU). *Ibid*., 13 June 2011, *Yaoundé shadehouse series 2865* (YA). *Ibid*., 1 September 2011, *Yaoundé shadehouse series 3075* (BRLU). *Ibid*., 7 May 2012, *Yaoundé shadehouse series 3550* (K, MO, WAG). Southwest Region, Nguti village, between the WCS station and the camp “552”, on a forestry trail at the border of the Banyang-Mbo Wildlife Sanctuary, 05.355°N, 09.491°E, 4 June 2017, *Droissart & Kamdem N. 2382* (BRLU, YA). Southwest Region, north of Ebonji village, on the trail to Mahole village, on cocoa tree, 04.773°N, 09.597°E, 8 June 2017, *Droissart & Kamdem N. 2407* (BRLU, YA). *Ibid.*, on kola tree, 8 June 2017, *Droissart & Kamdem N. 2409* (BRLU, YA). Southwest Region, northeast of Ebonji village, approximately 500 m from west bank Mungo River, on *Desbordesia glaucescens* (Engl.) Tiegh. (Irvingiaceae), 04.771°N, 09.578°E, 9 June 2017, *Droissart & Kamdem N. 2420* (BRLU, YA). REPUBLIC OF THE CONGO. Grand escarpement d’Odzala, bai de l’ombrette, forêt à *Gilbertiodendron dewevrei* (De Wild.) J.Leonard (Fabaceae), 01.067°N, 14.467°E, 1996, *Lejoly 96/1063* (BRLU).

##### Distribution.

**Figure 1.**
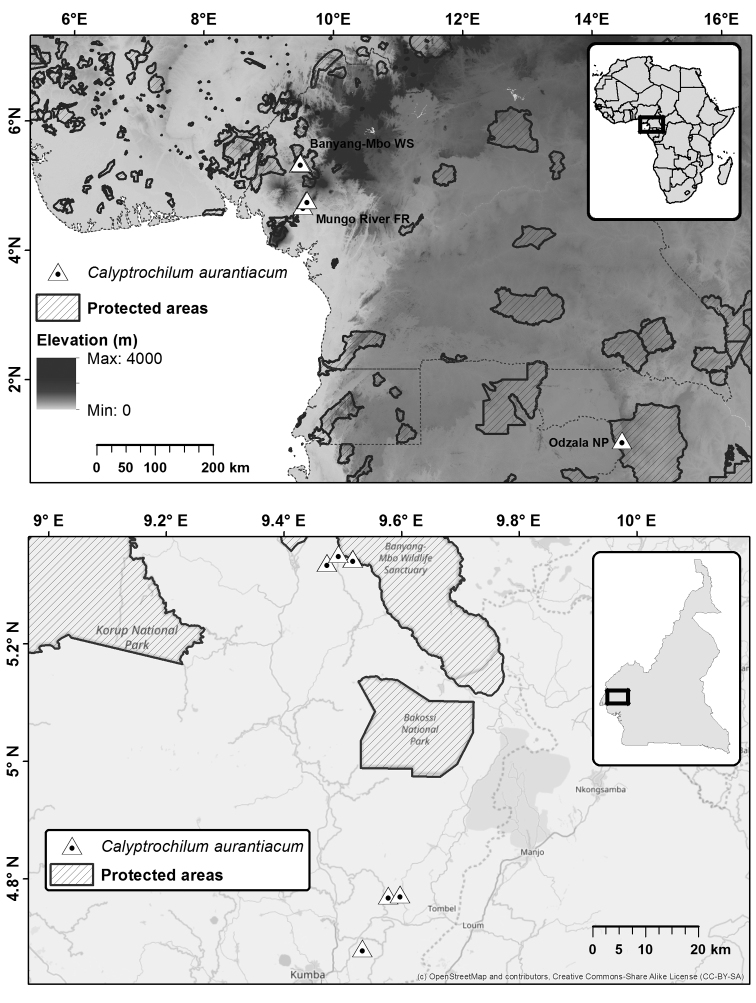
Current distribution of *Calyptrochilum aurantiacum* in Central Africa (upper map) and in Cameroon (lower map). WS = Wildlife Sanctuary; FR = Forest Reserve; NP = National Park.

Cameroon and the Republic of the Congo (Figure 1). This species is reported here for the first time in the Republic of the Congo. Its presence in a forest with *Gilbertiodendron dewevrei* suggests that *Calyptrochilum aurantiacum* might be more widely distributed, as this type of monodominant forest extends from Nigeria to the Democratic Republic of the Congo and northern Angola.

##### Ecology and habitat.

**Figure 2.**
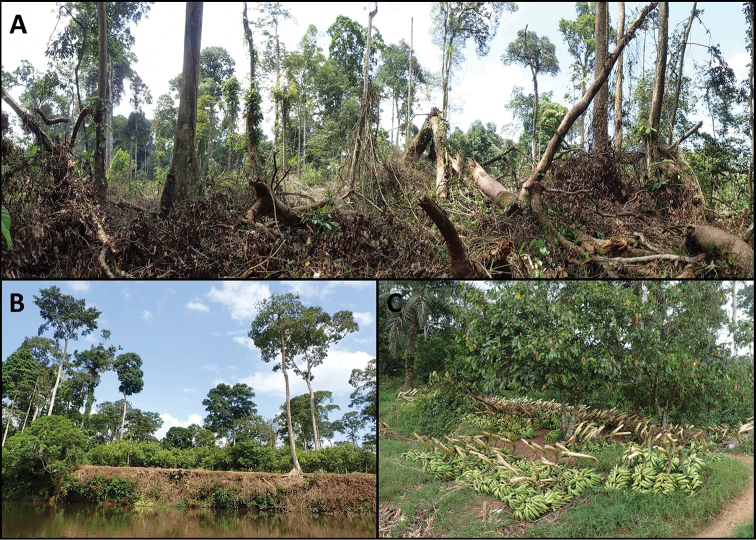
Habitat degradation and forest clearance at the type locality of *Calyptrochilum aurantiacum* (Mungo River Forest Reserve). In less than 10 years, hundreds of hectares of mature lowland rainforest have been converted to cocoa plantations along the Mungo River (pers. comm. by local communities). **A** Freshly cleared forest where plantain and bananas will be cultivated during two years prior to being replaced by cocoa trees. **B** A ten-year old cocoa plantation on the banks of the Mungo River; only a few shade trees remain from the original forest. **C** Plantain and banana bunches that are harvested every day along the Mungo River. Photographs by V. Droissart (June 2017).

*Calyptrochilum aurantiacum* occurs between 240 to 600 m elevation in mature dense lowland forest (sometimes degraded), in plantations and in *Gilbertiodendron dewevrei* monodominant forest. It was initially collected growing as an epiphyte 35 m above the ground on the lower branches of a primary forest tree (van der Laan and Cribb 1986). In 2017, we collected the species near the type locality on *Desbordesia glaucescens* (Irvingiaceae) and *Scottellia* sp. (Achariaceae) that had recently been felled to establish a new cocoa and banana plantation (Figure 2). In the same locality, the species was also collected on kola and cocoa trees in a 7-year old cocoa plantation. Within and around the Banyang-Mbo Wildlife Sanctuary, it was collected in the canopy of large trees (i.e. *Duguetia staudtii* (Engl. & Diels) Chatrou (Annonaceae) and *Klainedoxa gabonensis* (Pierre ex Engl.) (Irvingiaceae)).

**Figure 3.**
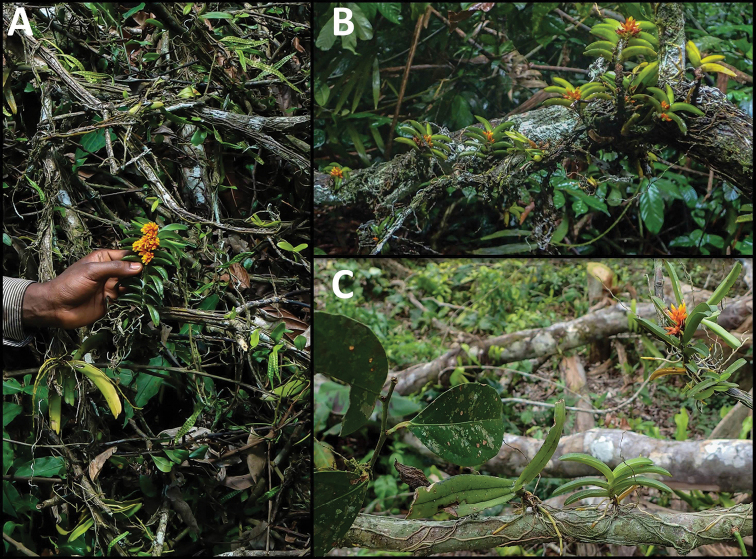
Ecology and habit of natural populations of *Calyptrochilum aurantiacum*. **A** Flowering individual growing on upper branches of a felled kola tree, along with the angraecoid orchid *Diaphananthe plehniana* and the fern *Microgramma owariensis*. **B** Dense population growing on 1 metre long branches of *Duguetia staudtii*. **C** *Calyptrochilum aurantiacum* and *Diaphananthe plehniana* growing side by side on *Salacia* sp. (Celastraceae). Photographs by V. Droissart (June 2017).

*Calyptrochilum aurantiacum* is a heliophile epiphyte growing on branches of 5 to 20 cm in diameter under the canopy of large trees (from 50 to more than 100 cm diameter at breast high). In most observed trees (ten observations), *Calyptrochilum aurantiacum* was found growing together with the angraecoid orchid *Diaphananthe plehniana* (Schltr.) Schltr. and with the fern *Microgramma owariensis* (Desv.) Alston (Polypodiaceae) (Figure 3). On *Duguetia staudtii* and *Desbordesia glaucescens*, we observed more than 100 individuals per tree. The flowering and fruiting of *C. aurantiacum* occur during the large rainy season, from May to November.

### Conservation

The extent of occurrence (EOO) of *Calyptrochilum aurantiacum* is estimated to be 19948.1 km^2^, which falls within the limits for “Vulnerable” (VU) category under criterion B1, whereas its area of occupancy (AOO) is estimated to be 28 km^2^, which falls within the limits for “Endangered” (EN) category under criterion B2. *Calyptrochilum aurantiacum* is an epiphyte of lowland evergreen rainforest. The species occurs within two officially protected areas: the Banyang-Mbo Wildlife Sanctuary in Cameroon and the Odzala National Park in Republic of the Congo. These two protected areas appear well managed. In other sites where *C. aurantiacum* occurs in Cameroon (within the Mungo River Forest Reserve and outside of the Banyang-Mbo Wildlife Sanctuary), its habitat is currently experiencing a great deal of human pressure (i.e. lowland forest is currently under threat from logging, farming practices, establishment of crop plantations, population growth and human migration, leading to urban expansion). From our recent observations made in June 2017 (Figure 2), the Mungo River Forest Reserve in Cameroon can no longer be considered as an effective protected area. Indeed, the recent construction of a road along the border of the Mungo River Forest Reserve provides greater accessibility, resulting in intensification of forest clearance inside and outside of the reserve’s boundary. The main threats to the species are timber exploitation in the north of the Banyang-Mbo Wildlife Sanctuary, cocoa, plantain and banana plantations in the Mungo River Forest Reserve and shifting agriculture and oil palm plantation on the eastern part of the Banyang-Mbo Wildlife Sanctuary. These activities are gradually transforming the species habitat and we think that this degradation will continue in the future.

There are 20 herbarium specimens of *Calyptrochilum aurantiacum* collected in six localities that represent three subpopulations: two in Cameroon (the Mungo River Forest Reserve and the Banyang-Mbo Wildlife Sanctuary) and the third in the Republic of the Congo. These three subpopulations represent five locations (*sensu* IUCN 2017), which fall within the limits for the IUCN category “Endangered” (EN) under criterion B2. The projected ongoing loss of its habitat leads us to predict a continuous decline in mature individuals of the species. Thus, *C. aurantiacum* is assigned an IUCN conservation status of EN B2ab(iii,v).

## Discussion

The integration of molecular evidence into current taxonomical practice and the adoption of a strictly phylogenetic Linnean framework in orchid classification have been altering many generic boundaries (Chase et al. 2015). For instance, two other African angraecoid genera, *Podangis* Schltr. and *Sphyrarhynchus* Mansf. considered monotypic, were recently recircumscribed and now comprise, respectively, two (Cribb and Carlsward 2012) and three species (Martos et al. 2017). Our recent molecular phylogeny on African angraecoid orchids (Simo-Droissart et al. 2018a) substantially improved the understanding of taxonomic relationships among the angraecoid orchids, leading us to describe a new genus to accomodate continental African species of Angraecum Bory sect. Pectinaria (Benth.) Schltr. (Simo-Droissart et al. 2018b) and to transfer *Ossiculum aurantiacum* to *Calyptrochilum*.

Our recent observations on the rapid degradation of the natural habitat of *Calyptrochilum aurantiacum* within the Mungo River Forest Reserve and around the Banyang-Mbo Wildlife Sanctuary both in Cameroon, stress the need for urgent actions to protect the last remaining patch of intact lowland forests from human activities. The survival of several other plant species endemic to this area, such as *Afrothismia winkleri* (Engl.) Schltr. (Burmanniaceae, IUCN status = CR, IUCN SSC East African Plants Red List Authority 2013), *Cola metallica* Cheek (Malvaceae, IUCN status = CR, Cheek 2003), *Tricalysia lejolyana* Sonké & Cheek (Rubiaceae, IUCN status = EN, Sonké et al. 2004), also relies on the conservation of these forests occurring below 600 m elevation.

**Figure 4.**
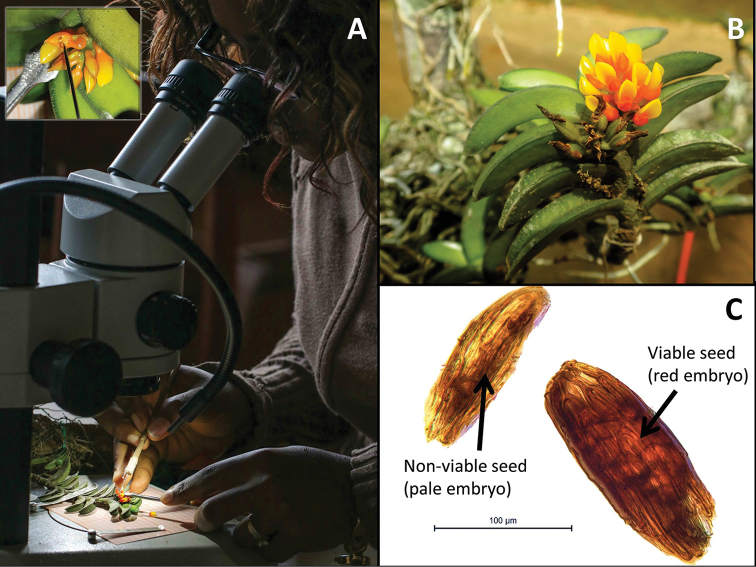
Seed banking of *Calyptrochilum aurantiacum* in Yaoundé (Cameroon). In 2017, an *ex situ* conservation programme was initiated to support the long term preservation of *C. aurantiacum*. **A** Due to the small size of the flowers, manual pollination has been performed under a stereomicroscope. **B** Fruit development and maturation takes place in a shadehouse established in Yaoundé. **C** Finally, viable seeds have been harvested and conserved in the freezer (-20°C). Before being preserved at low temperature, seed viability is assessed using the tetrazolium test (red colouration of living embryo). Photographs **A** and **C** by V. Droissart, B by Gyslène Kamdem.

Due to the ongoing destruction of its natural habitat, *Calyptrochilum aurantiacum* has been the focus of an *ex situ* conservation project. The species has been cultivated since 2004 in our *ex situ* collection in Yaoundé, Cameroon. Living specimens, collected from fallen branches and trees in the field, have been successfully grown and they have flowered for several consecutive years. Presently, about 150 living individuals are being cultivated in the shadehouse. Since November 2015, we have performed eight hand pollination experiments on *C. aurantiacum* in the shadehouse and the species has been introduced in our seed bank in Yaoundé (Figure 4). However, due to the small size of its flowers, manual pollination of *C. aurantiacum* is still challenging as the fruit set (resulting from hand self-pollination) remains low (~25%). On-site studies on reproductive biology of the species are thus needed to identify pollination mechanisms involved and to check if the pollinator(s) is/are also threatened by forest clearance.

The patchy distribution of *Calyptrochilum aurantiacum* seems surprising and suggests that additional surveys should be made to search for new populations. The discovery of the satellite population in the Republic of the Congo also raised the question whether the Cameroonian and Congolese populations of *C. aurantiacum*, separated by more than 650 km, have different species of pollinators. Seemingly, the intriguing pollinator may be absent in Yaoundé since we have never observed “natural” pollination on the living plants in cultivation.

Notwithstanding the success of our *ex situ* conservation programme, strategies to ensure the long-term survival of *C. aurantiacum* must focus primarily on *in situ* conservation efforts. During the 2017 survey, we found one individual growing on a cocoa tree within a plantation at Ebonji, Cameroon. Since clearance of forest for conversion to agricultural plantations will continue to increase, we performed in September 2017 the transplantation of 45 individuals on three cocoa trees (15 on each) in Ebonji village, in order to see if the species could maintain viable populations in those plantations. If the transplantation tests prove successful, the next step will be to ensure that the pollinator will also survive in this human-transformed habitat.

## Conclusion

Morphological and DNA-based evidence led to the transfer of the narrow-endemic *Ossiculum aurantiacum* to the widespread genus *Calyptrochilum* and, thus, recognises the monotypic genus *Ossiculum* as a junior synonym of *Calyptrochilum*. Thanks to recent intensive field trips and laboratory work on central African orchids, new localities of *Calyptrochilum aurantiacum* have been discovered, a situation that contributed to downgrade the IUCN conservation status of the species. *Calyptrochilum aurantiacum* is currently known from five locations in two countries (Cameroon and Republic of the Congo) and is assessed as Endangered [EN B2ab(iii,v)] according to the IUCN Red List Categories and Criteria. Fieldwork within and around the Odzala National Park is required to evaluate the species habitat and the state of the subpopulation there. *In situ* studies on reproductive biology would be necessary for greater efficiency in conservation measures.

## Supplementary Material

XML Treatment for Calyptrochilum

XML Treatment for Calyptrochilum aurantiacum
